# Creation of a new genus in the family *Secoviridae* substantiated by sequence variation of newly identified strawberry latent ringspot virus isolates

**DOI:** 10.1007/s00705-019-04437-0

**Published:** 2019-10-17

**Authors:** A. M. Dullemans, M. Botermans, M. J. D. de Kock, C. E. de Krom, T. A. J. van der Lee, J. W. Roenhorst, I. J. E. Stulemeijer, M. Verbeek, M. Westenberg, R. A. A. van der Vlugt

**Affiliations:** 1 Wageningen University and Research, P.O. Box 69, 6700 AB Wageningen, The Netherlands; 2 National Plant Protection Organization, P.O. Box 9102, 6700 HC Wageningen, The Netherlands; 3 Dutch Flower Bulb Inspection Service (BKD), P.O Box 300, 2160 AH Lisse, The Netherlands

## Abstract

To obtain insight into the sequence diversity of strawberry latent ringspot virus (SLRSV), isolates from collections and diagnostic samples were sequenced by high-throughput sequencing. For five SLRSV isolates, the complete genome sequences were determined, and for 18 other isolates nearly complete genome sequences were determined. The sequence data were analysed in relation to sequences of SLRSV and related virus isolates available in the NCBI GenBank database. The genome sequences were annotated, and sequences of the protease-polymerase (Pro-Pol) region and coat proteins (CPs) (large and small CP together) were used for phylogenetic analysis. The amino acid sequences of the Pro-Pol region were very similar, whereas the nucleotide sequences of this region were more variable. The amino acid sequences of the CPs were less similar, which was corroborated by the results of a serological comparison performed using antisera raised against different isolates of SLRSV. Based on these results, we propose that SLRSV and related unassigned viruses be assigned to a new genus within the family *Secoviridae*, named “*Stralarivirus*”. Based on the phylogenetic analysis, this genus should include at least three viruses, i.e., SLRSV-A, SLRSV-B and lychnis mottle virus. The newly generated sequence data provide a basis for designing molecular tests to screen for SLRSV.

**Electronic supplementary material:**

The online version of this article (10.1007/s00705-019-04437-0) contains supplementary material, which is available to authorized users.

## Introduction

Strawberry latent ringspot virus (SLRSV), an unassigned member of the family *Secoviridae* [21], was first identified in rosaceous plants by Lister in Scotland more than 50 years ago [11]. Since then, the virus has been reported to infect a broad range of plant species worldwide, including economically important crops such as *Fragaria, Rubus* and *Vitis* [4] and ornamental plants such as *Lilium* spp. (lily) [5, 18], *Narcissus* spp. [3], and *Rosa* spp. [8]. Many hosts infected by SLRSV remain symptomless [13]. However, yellow vein banding has been observed in several *Mentha* clones in the United States [15]. In addition, in New Zealand, Tang et al. [19] observed chlorotic spots on *Anemone* sp., chlorotic streaks and necrotic rings on *Impatiens walleriana*, chlorotic spots on *Rubu*s spp., chlorotic mottle on *Solanum muricatum*, and vein chlorosis on *Tibouchina* sp. Severe symptoms have been reported on strawberry plants when SLRSV was present in mixed infections with the criniviruses strawberry pallidosis-associated virus and beet pseudo-yellows virus [12]. SLRSV has been shown to be transmitted by the nematodes *Xiphinema coxi* and *X. diversicaudatum* and through seed in *Apium graveolens* (celery) [24], *Chenopodium quinoa*) [25], *Lamium amplexicaule* [14], *Lilium* spp. [23], *Mentha arvensis* [20], *Rubus idaeus* [14] and *Stellaria media* [14]. Recently, molecular characterisation and analysis of SLRSV isolates, including those from new hosts and geographic regions, revealed considerable diversity among isolates of this widespread virus [2, 12, 15, 19, 26].

SLRSV is a non-enveloped virus that forms icosahedral particles with a diameter of approximately 30 nm. The genome comprises two positive-sense ssRNA molecules with an approximate size of 7.5 kb for RNA1 and 3.8 kb for RNA2. Both have a 3’-terminal poly(A) tail and a genome-linked virus protein (VPg) covalently bound at the 5’ end. These RNA molecules are translated into two large polyproteins, which are subsequently cleaved by viral 3C-like proteinases. RNA1 codes for proteins involved in replication, i.e., protease cofactor (Pro-C), helicase (Hel), protease (Pro) and RNA-dependent RNA polymerase (Pol), whereas RNA2 codes for the movement protein (MP) and the large and small coat proteins (CPs). Based on the current genus demarcation criteria described in the 10th Report of the International Committee on Taxonomy of Viruses (ICTV), which include the number of genomic RNA segments, the number of CPs, and the clustering in phylogenetic trees based on aa sequence alignments of the conserved Pro-Pol region [21], SLRSV cannot be assigned to any of the genera within the family *Secoviridae*. The same holds for two tentative new members of the family *Secoviridae* that were recently reported in the Republic of Korea, i.e., lychnis mottle virus (LycMoV) from *Lychnis cognate* [28] and cnidium vein yellowing virus (CnVYV) from *Cnidium officinale* [27]. Phylogenetic analysis of both the Pro-Pol region and the CPs of these viruses revealed their close relationship to SLRSV, and these viruses are distinct from all other members of the family *Secoviridae*.

Despite its worldwide occurrence, SLRSV is a regulated pest in many countries. Lily bulbs (*Lilium* spp.) have to be free from SLRSV for export to several Southeast Asian countries. Since lily is a symptomless host, bulbs are routinely tested for SLRSV in the Netherlands. Testing is performed by double-antibody sandwich enzyme-linked immunosorbent assay (DAS-ELISA). However, testing of various sample sets with different antisera has produced positive but variable serological results. Moreover, the reverse transcription polymerase chain reaction (RT-PCR) test described by Tang et al. [19] failed to detect some isolates that tested positive in DAS-ELISA. This lack of concordance is most likely due to the diversity of the SLRSV isolates tested [26]. To gain more insight in the diversity of SLRSV sequences, high-throughput sequencing (HTS) was performed on eleven SLRSV isolates from different virus collections and also on a selection of isolates from diagnostic lily samples that reacted differently in serological and molecular tests.

In this paper, we report sequence data and subsequent phylogenetic analysis of 23 SLRSV isolates, which show that these isolates can be separated into at least three distinct clades. This provides evidence for assigning these isolates to three distinct species within the family *Secoviridae* [16, 21, 22]. Moreover, including two tentative members of this family, LycMoV and CnVYV, in the phylogenetic analysis supported the assignment of all three of these viruses to a new genus in the family *Secoviridae*, for which the name “*Stralarivirus*” is proposed.

## Materials and methods

### Virus isolates

Table 1 provides an overview of the SLRSV isolates used in this study. Lily isolates were obtained from diagnostic samples from the Dutch flower bulb inspection service (BKD) and selected on the basis of differences in positive serological reactions in routine DAS-ELISA.

**Table 1:** Overview of all isolates used in this study, including the origin of the isolates and the NCBI GenBank database assigned accession numbers of the sequences obtained from the various isolates. Diagnostic samples are bulbs from commercial crops received by the Dutch flower bulb inspection service for routine testing for which only RNA was available

Sample number	Original host	Lab host	Isolation information		Sample received from	Owner’s collection code		NCBI GenBank accession no.	
			year	country			Full length	RNA1	RNA2
NCGR MEN 454.001	*Mentha gentilis* L. ‘Variegata’	Nicotiana benthamiana	2004	USA	Robert Martin	Mentha_454	Yes	NC_006964	NC_006965
12-001_Lilium	*Lilium* sp. type: Oriental	Chenopodium quinoa	2012	The Netherlands	NPPO-NL	NPPO-NL 4206022	Yes	MF796973	MF796974
13-023_Lilium	*Lilium* sp. type: Oriental	-	2012	The Netherlands	Diagnostic sample		No	MF796975	MF796976
13-024_Lilium	*Lilium* sp. type: Asiatic	-	2013	The Netherlands	Diagnostic sample		No	MF796977	MF796978
14-001_Lilium	*Lilium* sp. type Oriental-Trumpet hybrid	-	2014	The Netherlands	Diagnostic sample		No	MF796979	MF796980
14-002_Lilium	*Lilium* sp. type Oriental-Trumpet hybrid	-	2014	The Netherlands	Diagnostic sample		No	MF796981	MF796982
14-007_Lilium	*Lilium* sp. type Oriental-Trumpet hybrid	-	2014	The Netherlands	Diagnostic sample		No	MF796983	MF796984
14-008_Lilium	*Lilium* sp. type: Oriental	-	2014	The Netherlands	Diagnostic sample		No	MF796985	MF796986
14-010-1_Lilium	*Lilium* sp. type: Asiatic	-	2014	The Netherlands	Diagnostic sample		No	MF796989	MF796990
14-010-2_Lilium	*Lilium* sp. type: Asiatic	-	2014	The Netherlands	Diagnostic sample		No	MF796987	MF796988
14-019_Lilium	*Lilium* sp. type: unknown	-	2014	The Netherlands	Diagnostic sample		No	MF796991	MF796992
14-034_Lilium	*Lilium* sp. type Oriental-Trumpet hybrid	-	2014	The Netherlands	Diagnostic sample		No	MF797007	MF797008
14-035_Lilium	*Lilium* sp. type Oriental-Trumpet hybrid	-	2014	The Netherlands	Diagnostic sample		No	MF797009	MF797010
17-007_Lilium	*Lilium* sp. type: Asiatic	-	2016	The Netherlands	Diagnostic sample		No	MG062675	MG062674
5875017_Lilium	*Lilium* sp. type Oriental-Trumpet hybrid	Chenopodium quinoa	2016	The Netherlands	NPPO-NL	NPPO-NL 5875017	Yes	MH237605	MH237606
14-021_Clematis	*Clematis* sp.	Nicotiana occidentalis P1	?	Norway	Dag-Ragnar Blystad	CleH92-4	No	MF796993	MF796994
14-023_Fragaria	*Fragaria* sp.	Chenopodium quinoa	1992	Germany	Jiří Svoboda	FPV19	Yes	MF796997	MF796998
14-024_Phaseolus	*Phaseolus vulgaris* “Narda”	Chenopodium quinoa	1978	The Netherlands	Wageningen University & Research	SLRSV_B143III	No	MF796999	MF797000
15-017_Prunus persica	*Prunus persica*	Chenopodium quinoa	2006	France	Denise Altenbach and Jean-Sebastien Reynard	5674880	Yes	MF797011	MF797012
14-027_Robinia	*Robinia pseudoacacia*	Nicotiana occidentalis P1	2009	Poland	Henryk Pospieszny	NPPO-NL 5895990*	No	MF797005	MF797006
14-025_Rosa rugosa	*Rosa rugosa*	Chenopodium quinoa	1991	The Netherlands	Wageningen University & Research	SLRSV_PD	Yes	MF797001	MF797002
14-026_Rosa	*Rosa* sp. “Betty Prior”	Chenopodium quinoa	1971	The Netherlands	Wageningen University & Research	SLRSV_R44	No	MF797003	MF797004
14-022_Rubus	*Rubus* sp.	Cucumis sativus	2005	New Zealand	Joe Tang	05/09	No	MF796995	MF796996
15-018_Rubus	*Rubus* sp.	Chenopodium quinoa	2001	Switzerland	Denise Altenbach and Jean-Sebastien Reynard	5674899	No	MF797013	MF797014

*Historic sample from NPPO-NL collection, owner’s collection code unknown

### RNA isolation and HTS

Total plant RNA was isolated from lily bulbs and symptomatic leaves of the test plants using an RNeasy Plant Mini Kit, including an on-column DNase treatment according the manufacturer’s protocol (QIAGEN, Hilden, Germany). The RNA concentration was measured using a Quant-iT RiboGreen RNA Reagent Assay Kit (Invitrogen, Life Technologies, Paisley, UK). Total RNA (50 ng to 1 µg) was used for preparation of a library suitable for Illumina HiSeq paired-end sequencing (TruSeq Stranded Total RNA sample Preparation Kit with Ribo-Zero Plant (Illumina Inc, San Diego CA, USA)). The final library was eluted in 30 μl of elution buffer. The quality of the library was analysed using a Bioanalyzer 2100 DNA 1000 chip (Agilent Technologies, Santa Clara CA, USA) and quantified using the Qubit quantitation platform using Quant-iT PicoGreen (Invitrogen, Life Technologies).

A total of 24 molecular identifier (MID)-labelled library samples were pooled in equimolar proportions and diluted to 6 pM for TruSeq Paired End v2 DNA clustering on a partial flow cell lane. Final sequencing was performed on a HiSeq 2500 platform using 126, 7, 126 flow cycles for sequencing paired-end reads. All steps for clustering and subsequent sequencing were carried out according to the manufacturer’s protocols. Reads were split per sample by corresponding MIDs using CASAVA 1.8 software (Illumina) with no mismatch in the MID region allowed. Library preparation of 12-001_Lilium was performed after purification of poly(A)-tailed RNA molecules. The entire sequencing procedure was carried out at Wageningen Plant Research (Wageningen, The Netherlands).

HTS of isolate 5875017_Lilium was performed at GenomeScan (Leiden, The Netherlands), using an NEBNext Ultra Directional RNA Library Prep Kit for Illumina RNA-Seq rRNA reduction (Ribo-Zero) and an Illumina NextSeq500 machine.

### Data analysis

Data analysis was performed using CLC Genomics Workbench 9.5.4 (QIAGEN, Hilden, Germany). After quality trimming (settings: quality limit 0.05%; short reads < 75 nucleotides [nt] and broken pairs were discarded), reads were used for *de novo* assembly. Contigs with a minimum length of 1 kb and/or a coverage of more than 1000 reads were subsequently analysed, using BLASTn [29] and BLASTx [1] to select the contigs with virus sequences. Open reading frames (ORFs) were identified on the basis of homology to those of published SLRSV sequences.

### Completion of genome sequences

For five SLRSV isolates, genome sequences, i.e. both RNA1 and RNA2, were completed from end to end (Table 1). The 3’UTR of each RNA molecule was determined by sequencing RT-PCR fragments generated using segment-specific forward primers corresponding to sequences located at the 3’ end of the ORF in combination with an oligo dT primer. The 5’UTR sequence of each RNA segment was determined using a Roche 5′/3′ RACE Kit according to the manufacturer’s protocol. In this case, the reverse primers were based on the 5’ region of the ORF of each RNA. Sanger sequencing of all amplicons that were generated was performed by Macrogen Europe (Amsterdam, The Netherlands).

The five complete annotated nucleotide (nt) sequences of SLRSV genomes and the coding sequences of additional partially sequenced isolates have been submitted to the NCBI GenBank database (see Table 1 for accession numbers).

### Phylogenetic analysis

All phylogenetic analysis was performed in MEGA 7. Alignments were made with ClustalW, and trees were calculated by neighbour joining with1000 bootstrap replicates [9]. In the first phylogenetic analysis of the Pro-Pol region, the amino acid (aa) sequence of the conserved domains between the CG motif of the 3C-like proteinase and the GDD motif of the polymerase [10, 21] of the five full-length RNA1 sequences of SLRSV and selected members of the family *Secoviridae* were used.

In addition, a phylogenetic analysis was performed for both the Pro-Pol region and the CPs (large and small CP together), using all available SLRSV nt and aa sequences in the NCBI GenBank database, and also including the SLRSV-related isolates of LycMoV and CnVYV [6, 27, 28].

### Serology

For serological comparison, SLRSV isolates were propagated on *Chenopodium quinoa*, *Cucumis sativus*, *Nicotiana benthamiana* or *Nicotiana occidentalis*-P1. Systemically infected leaves were collected up to 21 days post-inoculation and stored at - 80 °C. Test samples were prepared by grinding leaf material (1:10 (w/v)) in extraction buffer (0.02 M phophate-buffered saline (PBS) including 0.05% (v/v) Tween 20 (Sigma), 2.0% (w/v) polyvinylpyrrolidone (PVP, Sigma), 0.2% (w/v) bovine serum albumin (BSA, Sigma)). An uninfected sample and extraction buffer (blank) were included as negative controls. All samples (200 µl) were tested in two replicates.

DAS-ELISA was performed in MICROLON^®^ 96-well medium-binding plates (Greiner Bio-One, the Netherlands) using SLRSV antisera from Bioreba AG (Switzerland; lot no. 240639) and Prime Diagnostics (The Netherlands; lot no. S215106 [coating] and S21481 [conjugate]). The tests were performed following the manufacturer’s instructions with minor modifications. Coating antibodies were diluted 1000-fold in carbonate coating buffer (pH 9.6) and incubated overnight at 4 °C. Leaf homogenate was incubated overnight at 4 °C. Conjugate was diluted 1000-fold in extraction buffer and incubated for 3 h at 37 °C. The enzyme substrate 4-nitrophenylphosphate (pNPF) (Fluka, The Netherlands) was added at a concentration of 0.75 mg/ml in substrate buffer (pH 9.6) and incubated at 37 °C. After 2 h, the optical density values (OD) were measured using a microplate reader (BioRad iMark) with dual filters at wavelengths of 405 and 655 nm. Results were considered positive when the OD value of a given sample was more than twice the mean OD value of the uninfected control.

## Results and discussion

### Results of HTS and sequence analysis

HTS analysis showed that all samples suspected of SLRSV infection indeed contained SLRSV sequences. The number of Illumina reads obtained for each sample and the percentage of SLRSV reads are shown in Table S1. Additional virus sequences were found in six of the diagnostic lily samples. Samples 14-10-1 and 14-10-2 also contained lily virus A, sample14-008_Lilium contained Plantago asiatica mosaic virus, and samples 13-024_Lilium, 14-008_Lilium and 17-007_Lilium contained tulip virus X (data not shown).

For all 23 samples analysed, a nearly full-length SLRSV contig could be assembled. SLRSV contigs were selected using a BLASTn comparison to SLRSV sequences available in the NCBI GenBank database. Full-length sequences were generated by 5’-and 3’-RACE for five isolates, which were selected, either because they were used for antiserum production by Prime Diagnostics (14-025_Rosa_rugosa) and Bioreba (15-017_Prunus_persica) or because of their position in phylogenetic trees.

All of the newly generated SLRSV genome sequences showed a genome organisation that was identical to that of the only SLRSV isolate with full-length sequences published so far, (isolate NCGR MEN 454.001) from golden ginger mint [22]: two RNA segments, each containing one ORF encoding one large polyprotein, with RNA1 coding for the putative Hel, Pro-C, VPg, Pro, and Pol and RNA2 coding for the putative MP and two CPs. An overview of all of the viral genomes is given in Tables 1 and S3.

In comparison to the NCGR MEN 454.001 sequences (Table S3), the nt sequences of the ORFs of all ‘new’ SLRSV isolates show 69-87% identity in RNA1 and 66-95% identity in RNA2, and their deduced aa sequences show 78-95% identity in RNA1 and 72-98% identity in RNA2.

The SLRSV RNA1 polyprotein is predicted to code for five proteins, and the predicted dipeptide cleavage site S/G described previously for SLRSV [21] was present in all isolates. A comparison of the length of the proteins is presented in Table S3. The Hel, VPg, Pro and Pol sequences were the same length as those in the NCGR MEN 454.001 sequence. The length of the Pro-C, however, varied from 700 to 719 aa between isolates, and the Pol sequence of 15-017_Prunus_persica was found to contain one extra aa (asparagine) at the C-terminus. Of all of the proteins, the Pro-C sequences show the highest variation (58-91% identity) among the different isolates. The other proteins encoded by RNA1 were found to share 88-98% identity in Hel, 100% in VPg, 93-100% in Pro, and 86-97% in Pol.

The SLRSV RNA2 polyprotein is predicted to code for three proteins. All of the small CPs were the same length as those in the NCGR MEN 454.001 sequence (235 aa). For the MP and large CP, there was minor variation in length: 365-368 and 393-397 aa, respectively. The sequence of the 14-001_Lilium isolate contained a deletion of 816 nt (272 aa) in the MP-encoding region. To rule out an assembly artefact, this deletion was confirmed by RT-PCR with two primer sets recognizing sites on both sides of the missing region. The resulting amplicons were sequenced by the Sanger method, and these also showed the deletion. Due to this deletion, the MP of 14-001_Lilium shows only 22% sequence identity with the NCGR MEN 454.001 MP, whereas the other MPs show 87-98% identity. The LPL amino acid motif that is present in viral MPs [7] ws found in all of the sequences except 14-001 at position 184-186 or 183-185 (14-002_Lilium, 14-034_Lilium, 14-035_Lilium, 14-023_Fragaria, 15-017_Prunus_persica and 17-007_Lilium). The other proteins encoded by RNA2 were 69-98% and 52-97% identical in their large and small CP, respectively.

### Phylogenetic analysis

**Fig. 1:**
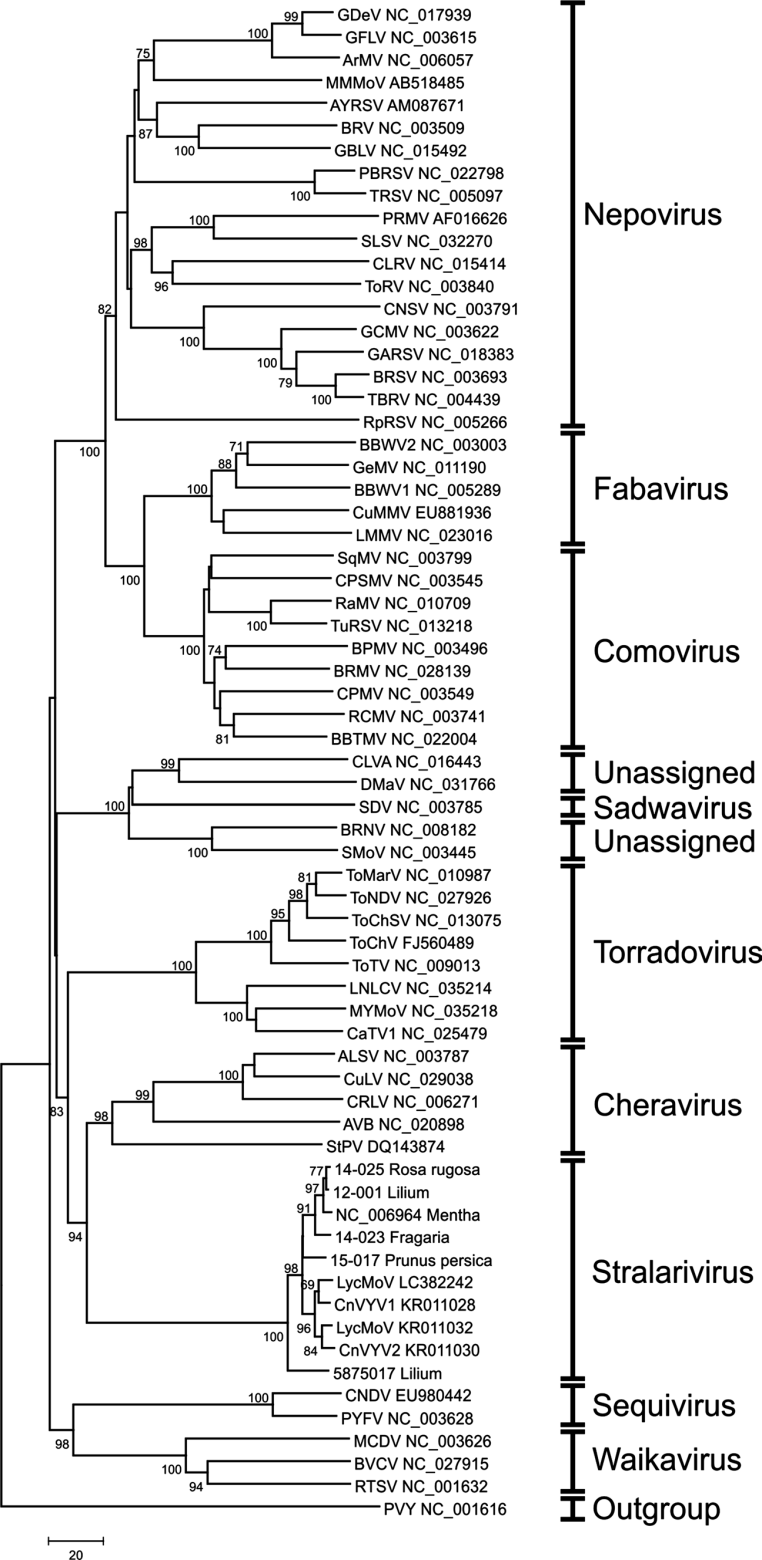
Rooted phylogram of the Pro-Pol aa region of the six full length SLRSV sequences and related members of the family *Secoviridae*, constructed using the neighbour-joining method. The percentage of replicate trees in which the associated taxa clustered together in the bootstrap test (1000 replicates) is shown next to the branches. Only values above 70% are shown. For a description of the virus abbreviations, see Table S6

Based on aa sequence comparisons of the Pro-Pol region of the full-length sequences of the SLRSV isolates and a selection of other members of different genera within the family *Secoviridae*, all of the newly generated SLRSV sequences grouped together with NCGR MEN 454.001 and isolates of the recently described viruses LycMoV, and CnVYV [6, 27, 28], which together formed a distinct clade (Fig. 1).

**Fig. 2:**
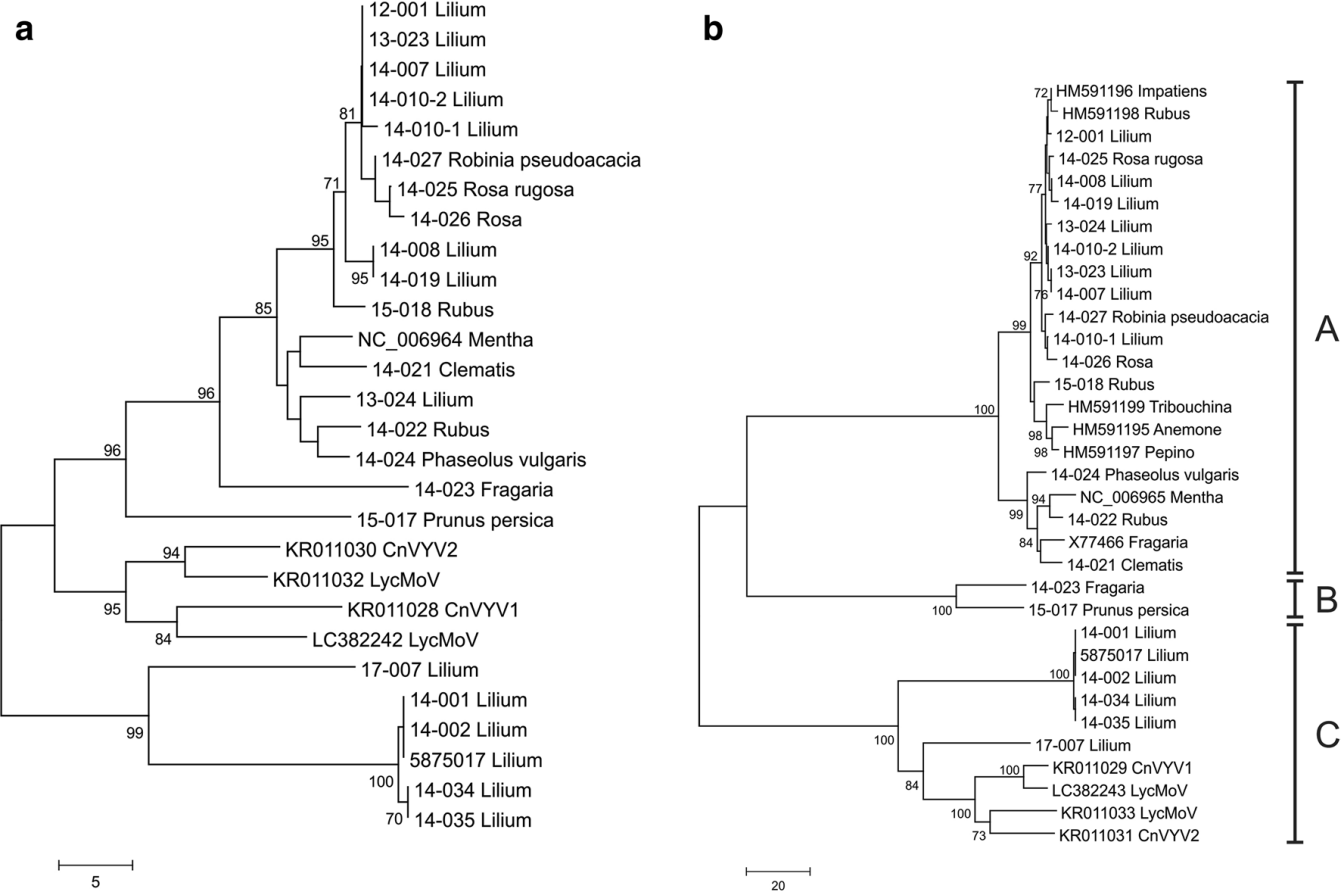
Unrooted phylogram of the Pro-Pol (**a**) and the CP (**b**) aa region of all available SLRSV and related sequences in the NCBI GenBank database, constructed using the neighbour-joining method. The percentage of replicate trees in which the associated taxa clustered together in the bootstrap test (1000 replicates) is shown next to the branches. Only values above 70% are shown

Phylogenetic analysis of the CPs show that the SLRSV isolates clustered in three distinct groups, referred to as groups A, B and C (Figs. 2b and S2b). Group A includes the isolates related to NCGR MEN 454.001 and the Prime Diagnostics antiserum isolate 14-025_Rosa_rugosa. Group B includes the Bioreba antiserum isolate 15-07_Prunus_persica and the 14-023_Fragaria isolate. Group C includes the six lily isolates 14-001, 14-002, 14-034, 14-035, 5875017 and 17-007 and the isolates LycMoV, LycMoV-J, CnVYV1 and CnVYV2 [6, 27, 28]. The grouping shown in the CP phylogenetic trees (Figs. 2b and S2b) is not fully supported by the phylogenetic trees based on the Pro-Pol region (Figs. 2a and S2a). All of the Pro-Pol sequences showed > 88% aa sequence identity.

Based on both the nt and aa phylogenetic trees based on the CPs (Figs. 2b and S2b), the Dutch lily isolates branch in separate groups. Lily isolate 17-007_Lilium appears to be more closely related to the LycMoV and CnVYV virus isolates than to the other lily isolates in group C. 17-007_Lilium was obtained from a *Lilium* Asiatic type, whereas the other *Lilium* isolates in group C were obtained from two different *Lilium* Oriental-Trumpet hybrid types. The *Lilium* SLRSV isolates in group A originated from different Asiatic, Oriental and Oriental-Trumpet hybrid types (Table 1). No correlation was found between the *Lilium* types and the grouping of the virus isolates.

### Comparison of serological reactions and CP sequences

**Fig. 3:**
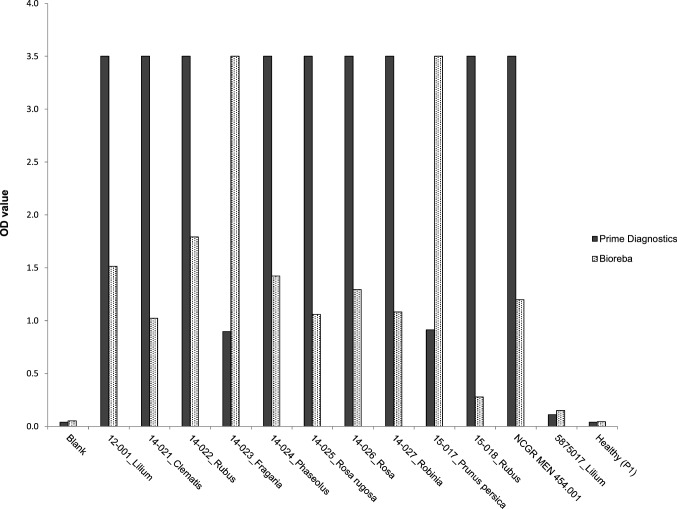
Results of DAS-ELISA (*y*-axis: mean OD value of duplicates) obtained for available SLRSV isolates (see Table 1), using antisera from Prime Diagnostics and Bioreba

One of the criteria for species demarcation in the family *Secoviridae* is serological relationship. For SLRSV, two antisera are commercially available from Prime Diagnostics and Bioreba, respectively. Testing a selection of available SLRSV isolates from groups A, B and C in DAS-ELISA showed different reactivity with each of the two antisera (Fig. 3). All tested isolates of group A, including isolate 14-025_Rosa_rugosa, against which the antiserum from Prime Diagnostics was raised, showed a strong reaction with the Prime Diagnostics antiserum and a modest-to-weak reaction with the Bioreba antiserum. In contrast, the isolates 15-017_Prunus_persica and 14-023_Fragaria of group B showed a strong reaction with the Bioreba antiserum, which was raised against isolate 15-017_Prunus_persica, and only a weak reaction with the Prime Diagnostics antiserum. The only available isolate of group C, i.e., 5875017_Lilium, reacted weakly with both antisera.

These distinct serological reactions between the three groups are corroborated by comparison to the phylogenetic tree of the aa sequences of the CPs (Fig. 2b and Table S4). Isolate 14-25_Rosa_rugosa (Prime Diagnostics antiserum) and 15-017_Prunus_persica (Bioreba antiserum) share only 71% identity at the aa level. In contrast, the CPs aa sequences of group A isolates share over 99% identity with isolate 14-25_Rosa_rugosa (and 92-100% between them) and only 70-71% identity with the two isolates from group B, which share 93% identity in their CPs. The aa sequences of group C share 81-100% identity but only 63-64% identity with 14-25_Rosa_rugosa and 65-66% identity with 15-017_Prunus_persica in their CPs, underlining the apparent clear serological distinction between groups A and B. The assignment of LycMoV and CnVYV to group C could not be confirmed serologically, because despite several attempts, it was not possible to obtain isolates of these viruses.

### Taxonomic position of SLRSV and related viruses

SLRSV is currently an unassigned member of the family *Secoviridae*. For this family, species demarcation is mainly based on the aa sequence of the Pro-Pol region (< 80% identity) and the aa sequence of the CPs (large and small CP together, < 75% identity) [21]. The recently described LycMoV and CnVYV [27, 28] show the highest sequence similarity to SLRSV within the family *Secoviridae.* LycMoV and CnVYV have been proposed as tentative members of the family *Secoviridae* solely based on the % aa sequence identity of the CPs to SLRSV sequences available in the NCBI GenBank database (LycMoV, 63%; CnVYV1, 64%; CnVYV2, 63%). These values are well below the current species demarcation limit of 75%. However, the % aa sequence identity values in the Pro-Pol region of these viruses to all SLRSV isolates analysed in this study are clearly above the demarcation threshold of 80% (LycMoV, 90%; LycMoV-J, 90%; CnVYV1, 88%; CnVYV2, 92%) [6, 27, 28], which would associate these isolates with SLRSV. A comparison between the aa sequences of LycMoV and CnVYV shows that they share over 94% identity in their Pro-Pol regions and 89-93% identity in their CPs, which would classify them as members of a single species within the family *Secoviridae*.

In addition to the comparisons of the Pro-Pol region and CPs and the differences in antigenic reactions, the ICTV describes several other species demarcation criteria for the family *Secoviridae*: distinct host range, distinct vector specificity, absence of cross-protection, and for viruses with a bipartite genome, absence of reassortment between RNA1 and RNA2. Not all criteria need to be met simultaneously [21].

In this study, the complete genome sequences of five SLRSV isolates and the nearly complete genome sequences of an additional 18 isolates were determined. With only one complete SLRSV sequence known so far, the new sequence data and analysis contributed significantly to the understanding of sequence diversity among SLRSV isolates and related unassigned members of the family *Secoviridae*. Comparisons of aa sequences of the Pro-Pol region and the CPs form an important basis for establishing taxonomic relationships within the family *Secoviridae*. The phylogenetic analyses show that all SLRSV isolates, and in addition the recently described LycMoV and CnVYV, form a distinct clade that is clearly separated from the other clades corresponding to genera within the family *Secoviridae* (Fig. 1 and Table S5). The overall level of sequence identity in the Pro-Pol region of the isolates in this SLRSV clade to the other clades/genera is just above 30% for the cheraviruses, just above 29% for the torradoviruses, and well below 30% for the other genera in the family *Secoviridae*. Therefore, based on the clearly distinct levels of sequence identity in the Pro-Pol region of all virus isolates in the SLRSV clade, the creation of a new genus, “*Stralarivirus*” (derived from “strawberry latent ringspot virus”) in the family *Secoviridae* is proposed.

So far, three distinct groups can be recognized within this newly proposed genus based on their CP sequences. The first group, designated group A, contains SLRSV isolates from a broad variety of plant species of different geographic origin. Nevertheless, these isolates share significant levels of nt and aa sequence similarity in their CPs, allowing them to be classified as isolates of a single virus species. The two isolates belonging to group B are sufficiently distinct from the isolates in group A in their CPs, with less than 75% aa sequence identity, that they can also be classified as isolates of a distinct species. This distinction is further supported by serological comparisons, showing a clear difference in reactivity in DAS-ELISA between two antisera raised against isolates from the respective groups A and B (see Fig. 3). Group C, comprising the two recently described tentative secoviruses LycMoV and CnVYV from medicinal plants from the Korean peninsula and Japan and six isolates from Dutch lilies, also qualifies for classification as a distinct species based on less than 65% aa sequence identity in their CPs to isolates from groups A and B. The limited serological data for this group – only one isolate was available for analysis – support this classification, but additional serological analyses of other group C isolates are desirable.

Based on their levels of sequence identity in their CPs, these three groups represent distinct species within the newly proposed genus “*Stralarivirus*”. However, this species grouping is not supported by the Pro-Pol regions, in which all show above 88% aa sequence identity.

For species demarcation, however, not all criteria have to always be met. The latest version of the ICTV report [21], available online on the ICTV website, states that in cases where the percentage of sequence identity in Pro-Pol and/or CP is near the proposed cutoff, other criteria should be considered and information on biological properties of the virus is useful. For example, beet ringspot virus (BRSV) and tomato black ring virus (TBRV) are considered members of separate species in the genus *Nepovirus.* Although they are closely related in their Pro-Pol sequences (89% aa sequence identity), they are much more divergent in their CP sequences (62% aa sequence identity). They differ in their antigenic reactions and also in the specificity of nematode transmission (BRSV is transmitted more efficiently by *Longidorus elongatus*, and TBRV is transmitted more efficiently by *Longidorus attenuatus*).

The situation for the isolates in the proposed genus “*Stralarivirus*” is analogous to the above example of BRSV and TBRV. The proposed species (groups A, B and C) are clearly distinguished based on their CP sequences, which is further supported by their serological reactivity. Unfortunately, not enough information about possible additional demarcation criteria is available; vectors have not (yet) been identified, and the host range does not separate the three groups.

Recently Shaffer et al. [17] reported the presence of LycMoV in peony in the United States, identified by HTS. Unfortunately, the full-length sequence of this isolate is not available, and it was therefore not possible to compare these data with those presented here. Shaffer et al. [17] presented five partial Pro-C sequences (MH878970 to MH878974) closely related to LycMoV from Korea (KR011032), with 74-75% nt sequence identity (84-85% aa sequence identity). Compared to the sequences reported in this paper, the nucleotide sequence identity in this region was 62-72%, 65-68%, and 68-73% (77-83%, 74-83% and 82-86% aa sequence identity) for group A, B and C respectively. Pro-C is the most variable protein in the SLRSV genome (Table S3), and no conclusions about the positioning of the LycMoV from peony within the proposed genus “*Stralarivirus*” can be made from the Pro-C comparisons.

In conclusion, the data presented here show that SLRSV and related isolates of unassigned members (LycMoV and CnVYV) of the family *Secoviridae* form at least three distinct groups in a separate newly proposed genus, “*Stralarivirus*”. Based on current species demarcation criteria for the family *Secoviridae* and additional serological comparisons, these groups can be considered members of three separate virus species within this newly proposed genus. The official names of these species still need to be determined. We propose to designate the virus isolates in group A as SLRSV-A and the isolates in group B as SLRSV-B, because group-specific antisera are currently available. The proposed virus name for the isolates in group C is “lychnis mottle virus” (LycMoV), as this was the first virus isolate described for this group. Finally, besides giving more insight into the variability of SLRSV, the newly generated sequence data may facilitate the design of more-reliable and better-distinguishing molecular tests for screening of routine samples for phytosanitary purposes.

## Electronic supplementary material

Below is the link to the electronic supplementary material.

Supplementary material 1 (DOCX 14 kb)

Supplementary material 2 (DOCX 430 kb)

Supplementary material 3 (DOCX 40 kb)

Supplementary material 4 (DOCX 544 kb)

Supplementary material 5 (DOCX 1998 kb)

Supplementary material 6 (DOCX 14 kb)
