# Supplementary material for: Creation of a new genus in the family Secoviridae substantiated by sequence variation of newly identified strawberry latent ringspot virus isolates
Source: Arch Virol. 2019 Oct 17;165(1):21–31. doi: 10.1007/s00705-019-04437-0 (PMC6954903; doi:10.1007/s00705-019-04437-0)
Supplement: Supplementary file 1 — Supplementary material 1 (DOCX 14 kb) [file 705_2019_4437_MOESM1_ESM.docx]

S1

Allocation of a new genus in the family *Secoviridae* substantiated by sequence variation of newly identified strawberry latent ringspot virus isolates.

Archives of Virology

authors: A.M. Dullemans, M. Botermans, M.J.D. de Kock, C.E. de Krom, T.A.J. van der Lee, J.W. Roenhorst, I.J.E. Stulemeijer, M. Verbeek, M. Westenberg, R.A.A. van der Vlugt

corresponding author: A.M. Dullemans: annette.dullemans@wur.nl

Number of Illumina reads per sample, the number of mapped reads per SLRSV RNA segment and the percentage of reads mapped on the SLRSV genome.

* : library preparation after purification of poly(A) tailed RNA.

| sample number | reads/sample | on SLRSV mapped reads | | % SLRSV reads | |  |
| --- | --- | --- | --- | --- | --- | --- |
|  |  | RNA1 | RNA2 |  |  |  |
| 12-001_Lilium | 31820064 | 13059605 | 18582009 | 99.4 * |  | |
| 13-023_Lilium | 18633624 | 5614 | 11996 | 0.1 |  | |
| 13-024_Lilium | 10404632 | 25411 | 69748 | 0.9 |  | |
| 14-001_Lilium | 12479010 | 730298 | 1113009 | 14.8 |  | |
| 14-002_Lilium | 23556490 | 899810 | 1455580 | 10.0 |  | |
| 14-007_Lilium | 25903822 | 1144923 | 3566338 | 18.2 |  | |
| 14-008_Lilium | 10131892 | 6371 | 7749 | 0.1 |  | |
| 14-010-1_Lilium | 15578692 | 1374834 | 2023960 | 21.8 |  | |
| 14-010-2_Lilium | 15895992 | 2073675 | 2179446 | 26.8 |  | |
| 14-019_Lilium | 12339496 | 662475 | 1659169 | 18.8 |  | |
| 14-034_Lilium | 7997614 | 228473 | 329979 | 7.0 |  | |
| 14-035_Lilium | 19738810 | 769732 | 1078581 | 9.4 |  | |
| 17-007_Lilium | 29353926 | 623286 | 326802 | 3.2 |  | |
| 5875017_Lilium | 29713912 | 53223 | 74669 | 0.4 |  | |
| 14-021_Clematis | 17416392 | 1504381 | 1478719 | 17.1 |  | |
| 14-023_Fragaria | 27675876 | 1470594 | 3713060 | 18.7 |  | |
| 14-024_Phaseolus | 23958184 | 1576857 | 2687779 | 17.8 |  | |
| 15-017_Prunus persica | 31641336 | 1058413 | 909419 | 6.2 |  | |
| 14-027_Robinia | 34206940 | 2907009 | 3722745 | 19.4 |  | |
| 14-025_Rosa rugosa | 18367818 | 2520452 | 3862450 | 34.8 |  | |
| 14-026_Rosa | 22166894 | 5555698 | 6510145 | 54.4 |  | |
| 14-022_Rubus | 38278916 | 983850 | 1233481 | 5.8 |  | |
| 15-018_Rubus | 25643376 | 879409 | 962402 | 7.2 |  | |
