# Supplementary material for: Creation of a new genus in the family Secoviridae substantiated by sequence variation of newly identified strawberry latent ringspot virus isolates
Source: Arch Virol. 2019 Oct 17;165(1):21–31. doi: 10.1007/s00705-019-04437-0 (PMC6954903; doi:10.1007/s00705-019-04437-0)

S2

Allocation of a new genus in the family *Secoviridae* substantiated by sequence variation of newly identified strawberry latent ringspot virus isolates.

Archives of Virology

authors: A.M. Dullemans, M. Botermans, M.J.D. de Kock, C.E. de Krom, T.A.J. van der Lee, J.W. Roenhorst, I.J.E. Stulemeijer, M. Verbeek, M. Westenberg, R.A.A. van der Vlugt

corresponding author: A.M. Dullemans: annette.dullemans@wur.nl

Unrooted phylogram of the Pro-Pol region(a) and the CPs (b) based on nt alignment of all available SLRSV sequences in NCBI GenBank. The percentage of replicate trees in which the associated taxa clustered together in the bootstrap test (1000 replicates) are shown next to the branches. Only values above 70% are shown.

Fig. S2a
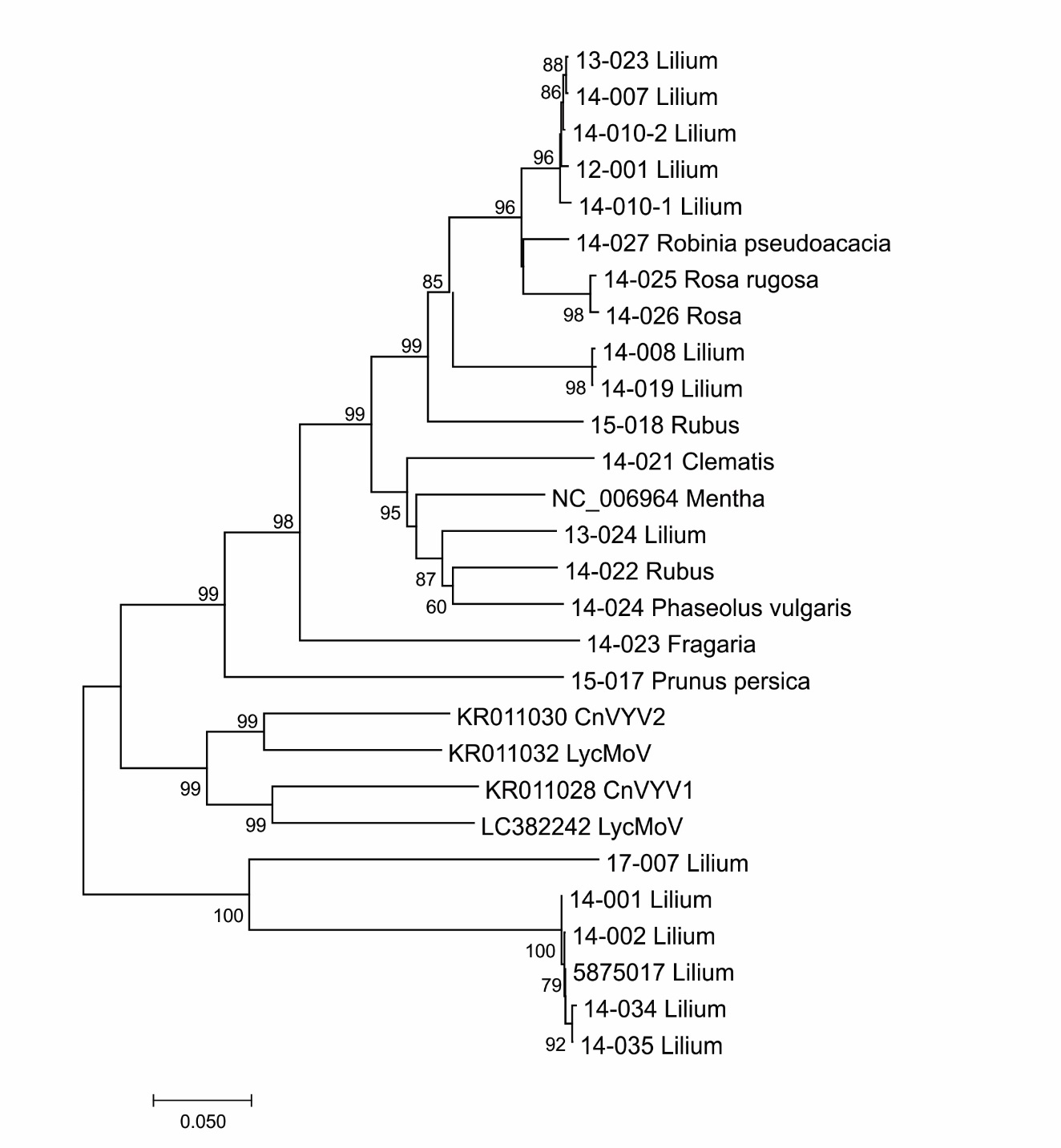


Fig. S2b
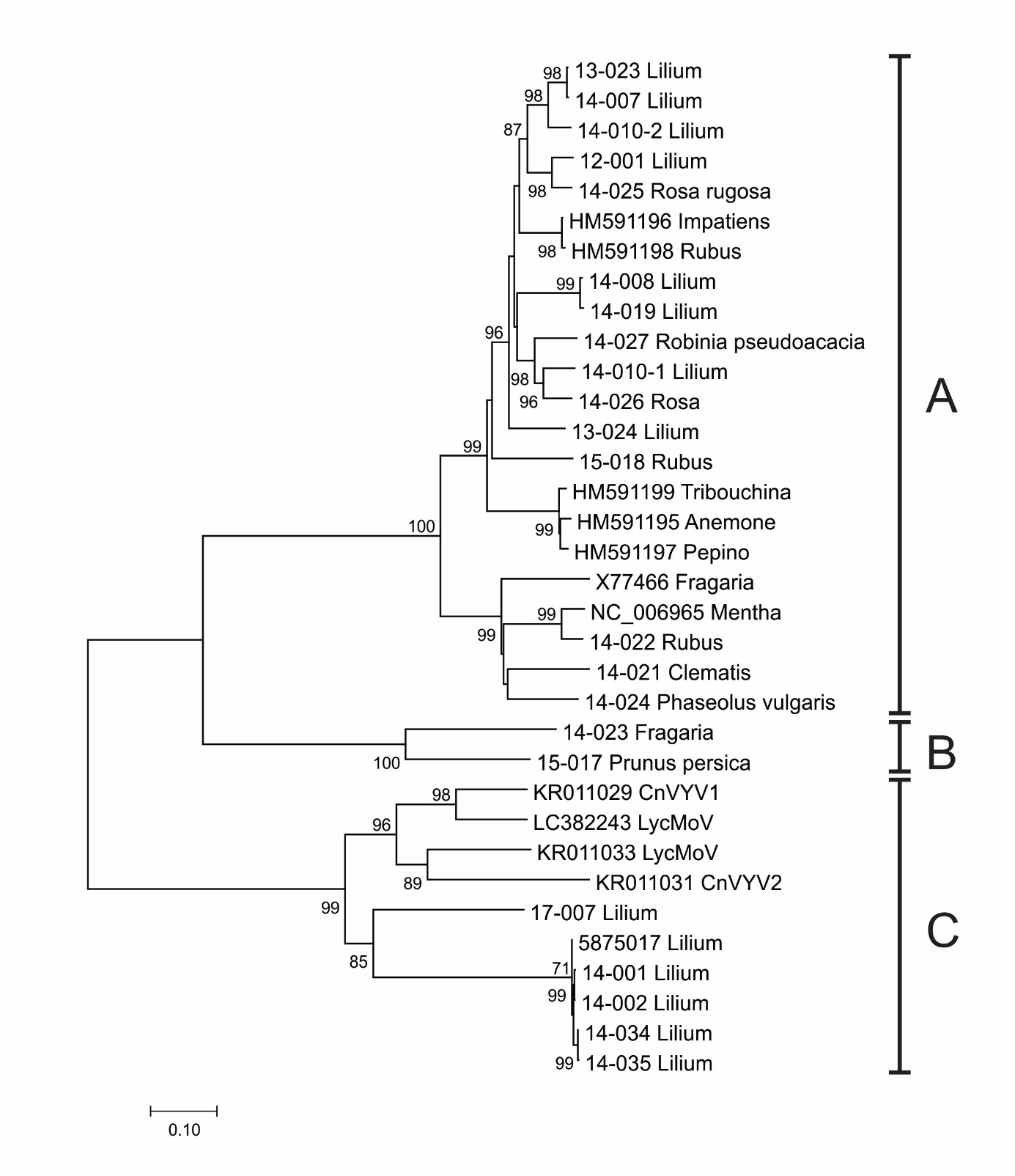

Supplement: Supplementary file 2 — Supplementary material 2 (DOCX 430 kb) [file 705_2019_4437_MOESM2_ESM.docx]
