# Supplementary material for: Creation of a new genus in the family Secoviridae substantiated by sequence variation of newly identified strawberry latent ringspot virus isolates
Source: Arch Virol. 2019 Oct 17;165(1):21–31. doi: 10.1007/s00705-019-04437-0 (PMC6954903; doi:10.1007/s00705-019-04437-0)
Supplement: Supplementary file 3 — Supplementary material 3 (DOCX 40 kb) [file 705_2019_4437_MOESM3_ESM.docx]

S3

Allocation of a new genus in the family *Secoviridae* substantiated by sequence variation of newly identified strawberry latent ringspot virus isolates.

Archives of Virology

authors: A.M. Dullemans, M. Botermans, M.J.D. de Kock, C.E. de Krom, T.A.J. van der Lee, J.W. Roenhorst, I.J.E. Stulemeijer, M. Verbeek, M. Westenberg, R.A.A. van der Vlugt

corresponding author: A.M. Dullemans: annette.dullemans@wur.nl

Overview of the genome organisation of all SLRSV isolates, including the full length sequences available in NCBI GenBank of SLRSV, CnVYV and LycMoV (shown in bold). The length of the UTRs and the proteins and the % of identity in nt and aa of the protein regions compared to the reference sequence are shown. Everything which resembles the SLRSV-NCGR MEN 454.001 genome organisation exactly is shaded. nc=not completed.
