# Supplementary material for: Creation of a new genus in the family Secoviridae substantiated by sequence variation of newly identified strawberry latent ringspot virus isolates
Source: Arch Virol. 2019 Oct 17;165(1):21–31. doi: 10.1007/s00705-019-04437-0 (PMC6954903; doi:10.1007/s00705-019-04437-0)

S5

Allocation of a new genus in the family *Secoviridae* substantiated by sequence variation of newly identified strawberry latent ringspot virus isolates.

Archives of Virology

authors: A.M. Dullemans, M. Botermans, M.J.D. de Kock, C.E. de Krom, T.A.J. van der Lee, J.W. Roenhorst, I.J.E. Stulemeijer, M. Verbeek, M. Westenberg, R.A.A. van der Vlugt

corresponding author: A.M. Dullemans: annette.dullemans@wur.nl

Levels of aa identity in the Pro-Pol region between viruses from the family *Secoviridae* corresponding to Fig. 1.

The table shows the % of aa identities. The comparisons are coloured by a gradient. The highest values are coloured dark red, and the lowest dark blue respectively.


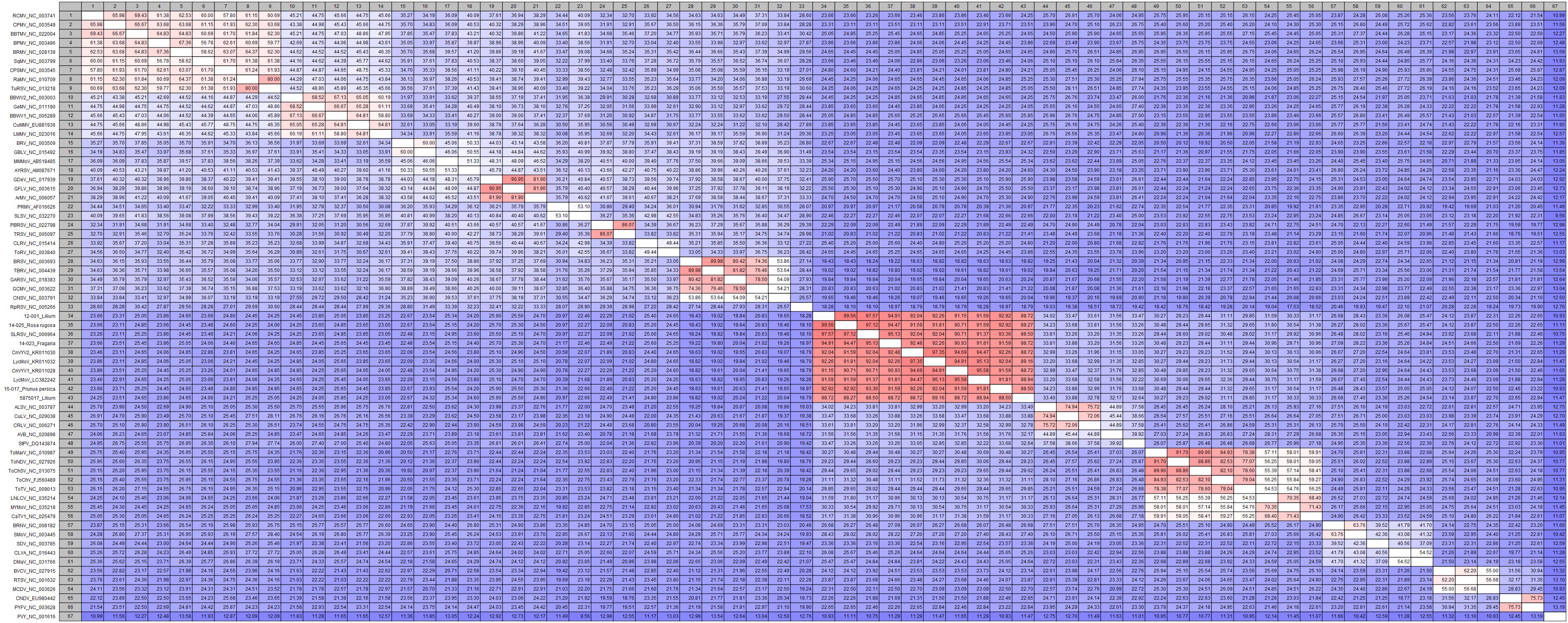

Supplement: Supplementary file 5 — Supplementary material 5 (DOCX 1998 kb) [file 705_2019_4437_MOESM5_ESM.docx]
