# Supplementary material for: Creation of a new genus in the family Secoviridae substantiated by sequence variation of newly identified strawberry latent ringspot virus isolates
Source: Arch Virol. 2019 Oct 17;165(1):21–31. doi: 10.1007/s00705-019-04437-0 (PMC6954903; doi:10.1007/s00705-019-04437-0)
Supplement: Supplementary file 6 — Supplementary material 6 (DOCX 14 kb) [file 705_2019_4437_MOESM6_ESM.docx]

S6

Allocation of a new genus in the family *Secoviridae* substantiated by sequence variation of newly identified strawberry latent ringspot virus isolates.

Archives of Virology

authors: A.M. Dullemans, M. Botermans, M.J.D. de Kock, C.E. de Krom, T.A.J. van der Lee, J.W. Roenhorst, I.J.E. Stulemeijer, M. Verbeek, M. Westenberg, R.A.A. van der Vlugt

corresponding author: A.M. Dullemans: annette.dullemans@wur.nl

Description of the virus acronyms used in the manuscript

| acronym | virus name | acronym | virus name |
| --- | --- | --- | --- |
| ALSV | apple latent spherical virus | LMMV | lamium mild mosaic virus |
| ArMV | arabis mosaic virus | LNLCV | lettuce necrotic leaf curl virus |
| AVB | arracacha virus B | LycMoV | lychnis mottle virus |
| AYRSV | artichoke yellow ringspot virus | MCDV | maize chlorotic dwarf virus |
| BBTMV | broad bean true mosaic virus | MMMoV | melon mild mottle virus |
| BBWV1 | broad bean wilt virus 1 | MYMoV | motherwort yellow mottle virus |
| BBWV2 | broad bean wilt virus 2 | PBRSV | potato black ringspot virus |
| BPMV | bean pod mottle virus | PRMV | peach rosette mosaic virus |
| BRMV | bean rugose mosaic virus | PVY | potato virus Y |
| BRNV | black raspberry necrosis virus | PYFV | parsnip yellow fleck virus |
| BRSV | beet ringspot virus | RaMV | radish mosaic virus |
| BRV | blackcurrant reversion virus | RCMV | red clover mottle virus |
| BVCV | bellflower vein chlorosis virus | RpRSV | raspberry ringspot virus |
| CaTV1 | carrot torradovirus 1 | RTSV | rice tungro spherical virus |
| CLRV | cherry rasp leaf virus | SDV | satsuma dwarf virus |
| CLVA | chocolate lily virus A | SLRSV | strawberry latent ringspot virus |
| CNDV | carrot necrotic dieback virus | SLSV | soybean latent spherical virus |
| CNSV | cycas necrotic stunt virus | SMoV | strawberry mottle virus |
| CnVYV | cnidium vein yellowing virus | SqMV | squash mosaic virus |
| CPMV | cowpea mosaic virus | StPV | stocky prune virus |
| CPSMV | cowpea severe mosaic virus | TBRV | tomato black ring virus |
| CRLV | cherry leaf roll virus | ToChSV | tomato chocolate spot virus |
| CuLV | currant latent virus | ToChV | tomato chocolate virus |
| CuMMV | cucurbit mild mosaic virus | ToMarV | tomato marchitez virus |
| DMaV | dioscorea mosaic-associated virus | ToNDV | tomato necrotic dwarf virus |
| GARSV | grapevine Anatolian ringspot virus | ToRV | tomato ringspot virus |
| GBLV | grapevine Bulgarian latent virus | ToTV | tomato torrado virus |
| GCMV | grapevine chrome mosaic virus | TRSV | tobacco ringspot virus |
| GDeV | grapevine deformation virus | TuRSV | turnip ringspot virus |
| GeMV | gentian mosaic virus |  |  |
| GFLV | grapevine fanleaf virus |  |  |
